# Lateral Trunk Motion and Knee Pain in Osteoarthritis of the Knee: a cross-sectional study

**DOI:** 10.1186/1471-2474-12-141

**Published:** 2011-06-29

**Authors:** Martin van der Esch, Martijn PM Steultjens, Jaap Harlaar, Josien C van den Noort, Dirk L Knol, Joost Dekker

**Affiliations:** 1 Reade, Center for Rheumatology and Rehabilitation, Department of Rehabilitation Research, Amsterdam, the Netherlands; 2 VU University Medical Center, Department of Rehabilitation Medicine, Department of Psychiatry, EMGO Institute, Amsterdam, the Netherlands; 3 VU University Medical Center, Department of Rehabilitation Medicine, Research Institute MOVE, Amsterdam, the Netherlands; 4 Glasgow Caledonian University, School of Health, Glasgow, Scotland UK

**Keywords:** Osteoarthritis, Knee, Trunk Motion, Knee Pain

## Abstract

**Background:**

Patients with osteoarthritis of the knee may change their gait in an attempt to reduce loading of the affected knee, thereby reducing pain. Especially changes in lateral trunk motion may be potentially effective, since these will affect the position of the centre of mass relative to the knee, enabling minimization of the load on the knee and thereby knee pain. The aim of the study was to test the hypothesis that a higher level of knee pain is associated with higher lateral trunk motion in patients with knee OA.

**Methods:**

Fifty-two patients with OA of the knee were tested. Lateral trunk motion was measured during the stance phase of walking with an optoelectronic motion analysis system and a force plate. Knee pain was measured with the VAS and the WOMAC pain questionnaire. Regression analyses were performed to assess the relationship between lateral trunk motion and knee pain.

**Results:**

It was shown that in bivariate analyses knee pain was not associated with lateral trunk motion. In regression analyses, pain was associated with more lateral trunk motion. In addition, more lateral trunk motion was associated with younger age, being female, higher self-reported knee stiffness and higher maximum walking speed.

**Conclusion:**

Pain is associated with lateral trunk motion. This association is weak and is influenced by age, gender, self-reported stiffness and maximum walking speed.

## Background

Osteoarthritis (OA) of the knee is characterized by changes in gait kinematics. These changes include reduced walking speed [1-3] shortening of stride length [2,4], reduced pelvic rotation [5] and increased lateral trunk motion [6-8]. The most distinct clinical sign is pain in or around the osteoarthritic knee. In an attempt to reduce pain, patients will try to reduce the load on the knee joint [9]. One of the available means is a change in gait pattern. Changes in gait kinematics (i.e. reduced walking speed, shortening of stride length, reduced pelvic rotation and increased lateral trunk motion) will influence knee loading and these gait changes are therefore related to knee pain during walking. Alterations in gait kinematics may be secondary, compensatory changes adopted by patients to lessen the load on the affected OA knee. Changes in lateral trunk motion may be potentially effective, seen that these will affect the position of the centre of mass relative to the knee, enabling minimization of frontal plane net joint moments on the knee and thereby knee pain [7,10]. Knowledge regarding the association between lateral trunk motion and knee pain is scarce.

**Figure 1:**
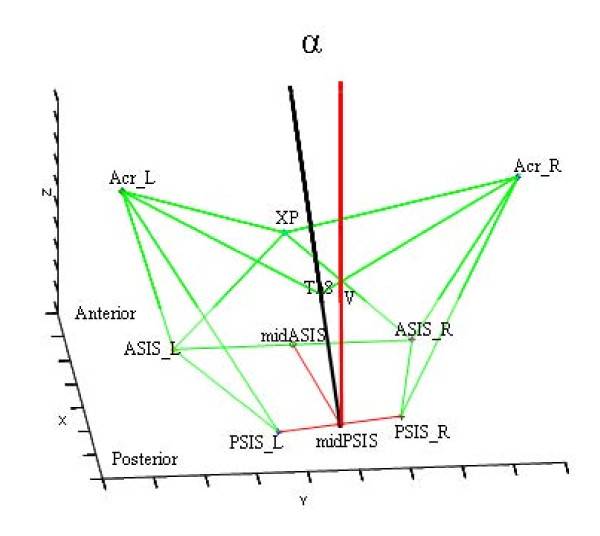
**Trunk orientation**. Trunk orientation was defined by the black line from the point halfway between the superior posterior iliac crest markers to the spinal processus thoracic 8. The red line (V) represents the line perpendicular to the plane of the pelvis (pointing upward). Lateral trunk angle α refers to the angle between the two lines, projected in the frontal plane. PSIS = posterior superior iliac spina. ASIS = anterior superior iliac spina. XP = xiphoid processus. Acr = acromion. V = vertical line.

Lateral trunk motion describes the lateral motion of the trunk in the frontal plane during walking. In this study lateral trunk motion was defined as the angular deviation in the frontal plane from the global vertical axis with the axis connecting the midpoint of the trans-acromion line and the midpoint of the trans-posterior-superior-spine (Figure 1). Increased lateral trunk motion results in a greater laterally deviated trunk over the limb at midstance. This is thought to reduce the knee adduction moment, and potentially the medial joint forces, which in turn may result in decreased knee joint pain [6-8]. However, the association between lateral trunk motion and knee pain was found to be rather weak (r = -0.18) [8]. An explanation for this weak association was not given. Therefore, the possible association between knee pain and lateral trunk motion in knee OA needs to be substantiated. This study aimed to test the hypothesis that a higher level of knee pain is associated with higher lateral trunk motion in patients with knee OA.

## Methods

Data from earlier studies on knee varus-valgus motion during gait were used [11,12]. Sixty-three patients diagnosed with OA of the knee were included in the study. Inclusion criteria were OA of the knee (uni- or bilateral) according to the clinical criteria of the American College of Rheumatology [13], and being between 40 and 85 years of age. Exclusion criteria were poly-arthritis, presence of rheumatoid arthritis or other systemic inflammatory arthropathies, knee surgery within the last twelve months or a history of knee arthroplastic surgery, intra-articular corticosteroid injections into either knee within the previous three months, and/or inability to understand the Dutch language. Four patients with BMI over 40 kg/m^2 ^were excluded due to difficulty in locating anatomical landmarks for proper marker placement. Complete gait analyses data were missing from seven patients. Therefore, the data from 52 patients were used in the analysis. All patients provided written informed consent. The human research ethics committee of the VU University Medical Center in Amsterdam approved the study.

Patients visited the laboratory twice within a week. During the first visit, patients completed a questionnaire, muscle strength was tested and a walking test was completed. The second visit consisted of a three-dimensional gait analysis.

A series of demographic variables were obtained including age, gender, height, weight, and duration of complaints.

Weight-bearing, anteroposterior radiographs of the knee joints were obtained following the Buckland-Wright protocol [14]. Radiographs of the knee were scored in a blinded fashion by an experienced radiologist using the grading scales proposed by Kellgren & Lawrence (K/L) [15,16]. Additionally, Joint Space Narrowing (JSN) was assessed as the interbone distance at the narrowest points of the medial and lateral tibia femoral compartments. A 4-grade 0[1-3] scale was used: (0 = no JSN; 1 = minute JSN; 2 = definite JSN; 3 = ankylosis). The scores were dichotomized as the presence [1-3] or absence (0) of JSN. Osteophytes were assessed with a similar rating scale (0 = no osteophyte; 1 = minute osteophyte; 2 = definite osteophyte, moderate size; 3 = large osteophyte). The scores were dichotomized as the presence [1-3] or absence (0) of osteophytes.

An Optotrak motion analysis system (model 3020, Northern Digital Inc., Waterloo, Ontario, Canada) recorded the 3D position of marker trajectories during walking at 50 Hz sampling frequency. A cluster of three markers (small infrared light emitting diodes (LEDs) were secured to each body segment. For this study, only the motions of the pelvis (cluster at the sacrum) and the trunk (cluster at the spinal processus C7) were considered. These clusters were anatomically calibrated probing the superior anterior and posterior iliac crest for the pelvis, and the acromion, the thoracic spinal processus 8 (Th8) and the xiphoid processus for the trunk [17]. To describe skeletal movement, body segments were considered as rigid bodies (pelvis and trunk) with a local coordinate system defined to coincide with a set of anatomical axes [17]. An open source Matlab software program BodyMech (http://www.bodymech.nl) was used to reconstruct the anatomical axes of the pelvis and trunk orientation [18]. The anatomical axis of the trunk was defined by the line from the point halfway between the left and right superior posterior iliac crest markers to the thoracic spinal processus 8 (Th8) (see Figure 1). Lateral trunk angle was expressed as the angle α between this line and a line perpendicular to the plane of the pelvis (pointing upward), projected in the frontal plane.

Ground reaction forces were recorded synchronously during walking with a sample frequency of 1000 Hz using a 51 × 46.5 cm force plate (AMTI, Watertown, Massachusetts, USA) to detect the stance phase (initial contact until toe off).

**Figure 2:**
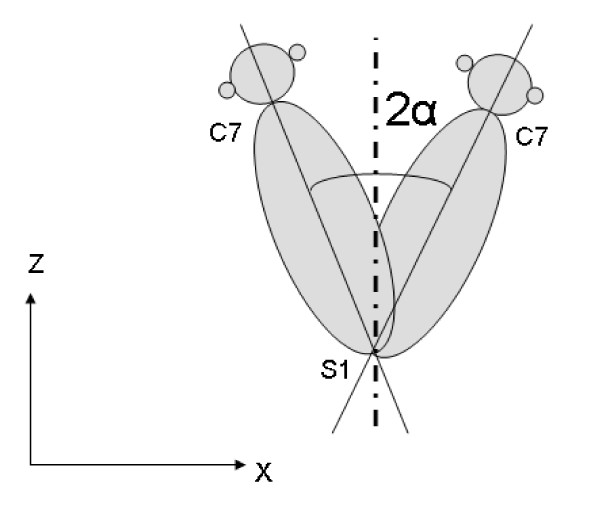
**Trunk motion**. Measurement of lateral trunk motion, expressed as the total angle 2α in degrees (i.e. the sum of the movement to the right and left) in the frontal plane (x, z). C7 represents the spinal processus of cervical 7 and S1 represents the spinal processus of sacral 1.

During walking all patients were instructed to walk at a self-selected speed along an 8 m walkway. In order to achieve a natural gait pattern, patients were not informed of the need to hit the force plate. The measurement was repeated when patients did not hit the force plate. The marker data and ground reaction forces from three accurate walking trials (i.e. hit on the force plate) were collected for both left and right knee (six in total). Trunk motion was defined by the minimum to maximum trunk angle (i.e. the range) during the stance phase on the force plate. The correlation between left and right trunk motion was high (r = 0.90; P < 0.001). Therefore, the sum score of the right and left trunk motion was used in analyses to obtain a measure for total trunk movement at the patient level (the angle 2 α; see Figure 2).

Knee-pain was measured on a Visual Analogue scale (VAS) (range, 0-10). A higher score on the VAS equates to a higher level of pain. The Dutch version of the Western Ontario and McMaster Universities Osteoarthritis Index (WOMAC) was used to assess self-reported pain. The WOMAC is a disease specific measure of pain and stiffness, for individuals with OA of the knee (the WOMAC physical function was also assessed, but we did not use that score in the present study). The WOMAC, with a possible range of 0-96, includes 5 items related to pain, 2 items related to stiffness, and 17 items related to physical function (PF). Each item is scored on a 5-point Likert scale. High scores on the WOMAC pain domain indicate a higher level of pain. Reliability and validity of the WOMAC have been established [19].

Maximum walking speed was calculated from a test that required patients to walk as fast as possible five times continuously up and down a level 20-meter corridor. A stopwatch was used to measure the time it took to complete the 100-meter distance, commencing from a verbal cue to start walking to culmination of the 5^th ^pass. Maximum walking speed in m/s during this walking test was used in analyses.

Muscle strength was measured isokinetically as has been described in previous studies [11,12,20,21]. The mean in Nm per kg body weight (Nm/kg) obtained from three measurements of the right and left maximum voluntary contraction of quadriceps and hamstrings were used for analysis. The mean of the right and left knee were averaged to obtain a measure for total muscle strength around the knee at the patient level [22]

### Statistics

First, Pearson correlation coefficients assessed the bivariate association between lateral trunk motion and knee pain. Additionally, the correlation coefficients were calculated between lateral trunk motion and knee pain and gender, age, stiffness (WOMAC), maximum walking speed, body mass index (BMI), duration of complaints, muscle strength and disease severity (K&L grade). Secondly, regression analyses were performed to assess the association between lateral trunk motion and knee pain. Regression analyses were repeated with adjustment for gender, age, stiffness (WOMAC), maximum walking speed, body mass index (BMI), duration of complaints, muscle strength and disease severity (K&L grade). These variables were included in analyses when they were found to be significant in bivariable analyses, known as common confounders or have been mentioned in literature as potentional confounder. When repeating the regression analyses each variable was entered one at a time (the enter method). The impact of the independent variables on lateral trunk motion was determined from the unstandardized and standardized regression coefficient, b and B. When these independent variables changed the regression coefficient of knee pain VAS or knee pain WOMAC for 10% then these variables were included in the regression model [23]. The significance level for exclusion from the final regression model was set at *P *< 0.05: regression coefficients were considered to be significant at *P *< 0.05. All analyses were performed using SPSS version 17.0 software (Chicago, IL).

## Results

Patient characteristics of 52 patients of the study sample are presented in Table 1. Mean ± SD lateral trunk motion was 22.7° ± 6.6° and knee pain (VAS) and knee pain (WOMAC) were 5.0 ± 2.4 and 11.29 ± 5.47, respectively.

**Table 1:** Characteristics of patients with knee osteoarthritis (N = 52)

		Mean ± SD	Range	N(%)
Gender	Female			43 (83%)
	Male			9 (17%)
Age, years		60.5 ± 8.0	45 - 79	
Body mass index, kg/m^2^		29.0 ± 3.9	22.4 - 37.4	
Duration of symptoms, years		6.2 ± 8.1	1 - 47	
Lateral trunk motion, degrees		22.7 ± 6.6	10.7 - 38.9	
Maximum walking speed (m/s)		1.14 ± 0.28	0.51 - 1.64	
WOMAC-Stiffness score		4.1 ± 1.9	0 - 8	
WOMAC-Pain score		11.2 ± 5.4	0 - 20	
Pain (VAS 0-10)		5.0 ± 2.5	0.3 - 9.3	
Isokinetic muscle strength, (quadriceps/hamstrings) Nm/kg		0.78 ± 0.35	0.08 - 1.63	
				
K&L grade*, no of knees				
Right				
	Grade 0			0
	Grade 1			37 (71.2%)
	Grade 2			8 (15.4%)
	Grade 3			5 (9.6%)
	Grade 4			1 (1.9%)
	Missing			1 (1.9%)
Left				
	Grade 0			2 (3.8%)
	Grade 1			33 (63.5%)
	Grade 2			7 (13.3%)
	Grade 3			8 (15.4%)
	Grade 4			2 (3.8%)
Joint Space Narrowing				
Right				
Medial				25 (48.0%)
Lateral				4 (7.6%)
Left				
Medial				29 (55.8%)
Lateral				9 (17.3%)
Osteophytes				
Right				
Medial				49 (94.3%)
lateral				44 (84.7%)
Left				
Medial				50 (96.2%)
Lateral				44 (84.6%)

WOMAC = Western Ontario and McMasters Universities Osteoarthritis Index

*Kellgren & Lawrence score of knee OA

In bivariable analyses, lateral trunk motion did not correlate significantly with knee pain VAS (r = 0.24; p = 0.09), knee pain WOMAC (r = 0.19; p = 0.17), gender (r = 0.16; p = 0.26), maximum walking speed (r = 0.21; p = 0.15), BMI (r = 0.07; p = 0.60), duration of complaints (r = -0.21; p = 0.14) or muscle strength (r = 0.01; p = 0.95). Lateral trunk motion was also not correlated with K&L severity and WOMAC-pf (r = 0.14; p = 0.28, r = 0.02; p = 0.85, respectively). Significant correlations were found for WOMAC stiffness (r = 0.32; p = 0.02) and age (r = -0.45; p = 0.00). Pain VAS and pain WOMAC were found to be correlated (r = 0.82; p = 0.00).

In multivariable analyses, the relationship between lateral trunk motion and knee pain was evaluated in a multiple linear regression analysis. The regression model of the relationship between lateral trunk motion and knee pain VAS and the regression model between lateral trunk motion and WOMAC pain are presented in Table 2 and 3. Regression analyses demonstrated that knee pain VAS is associated with lateral trunk motion (b = 0.77, p = 0.03). However, this relationship was only significant when age, gender, stiffness and maximum walking speed were also included in the regression model. This means that younger, female patients, self-reporting stiffness and capable of walking with a higher maximal walking speed during a maximal walking speed test had greater lateral trunk motion.

**Table 2:** Results of the regression of lateral trunk motion on knee pain (VAS), gender, age, stiffness (WOMAC) and maximum walking speed

Variables	b (SE)	Beta	p-value
Intercept	14.71		
Pain (VAS)	0.77 (0.35)	0.31	0.03
Gender (female vs male)	6.48 (2.00)	0.39	0.00
Age (years)	-0.27 (0.11)	-0.32	0.02
Stiffness (WOMAC)	1.05 (0.43)	0.30	0.02
Maximum walking speed (m/s)	9.59 (3.54)	0.41	0.01
	R^2 ^= 0.47 p < 0.001		

(N = 52)

b = unstandardized regression coefficient

SE = Standard Error

Beta = standardized regression coefficient

VAS = Visual Analogue Scale

WOMAC = Western Ontario and McMaster Universities Osteoarthritis Index

**Table 3:** Results of the regression of lateral trunk motion on knee pain (WOMAC), gender, age, stiffness (WOMAC) and maximum walking speed

Variables	b (SE)	Beta	p-value
Intercept	17.12		
Pain (WOMAC)	0.27 (0.17)	0.23	0.12
Gender (female vs male)	6.18 (2.04)	0.36	0.00
Age (years)	-0.28 (0.12)	-0.34	0.02
Stiffness (WOMAC)	1.12 (0.44)	0.32	0.01
Maximum walking speed (m/s)	9.03 (3.74)	0.38	0.02
	R^2 ^= 0.44 p < 0.001		

(N = 52)

b = unstandardized regression coefficient

SE = Standard Error

Beta = standardized regression coefficient

WOMAC = Western Ontario and McMasters Universities Osteoarthritis Index

WOMAC pain was not found to be a predictor of lateral trunk motion (b = 0.27, p = 0.12), even after adjusting for the same four confounders Other variables, e.g. duration of complaints, BMI and muscle strength did not change the regression coefficient of knee pain and contributed not to the variance in lateral trunk motion.

Additional analyses showed that the interaction terms between knee pain and the variables age, gender, stiffness and maximum walking speed were not significantly associated with lateral trunk motion.

## Discussion

This study showed that in bivariable analyses a higher level of pain is not associated with high lateral trunk motion during walking in patients with knee OA. However, multivariable analyses showed that knee pain was indeed weakly associated with more lateral trunk motion when age, gender, self-reported stiffness and maximum walking speed were accounted for.

The weak relationship between lateral trunk motion and knee pain, found in this study, is in agreement with Hunt et al [8]. However, the relationship found by Hunt et al was established using bivariable analyses, whereas in our study the association was only found in multivariable analyses. It seems that age, gender, stiffness and maximum walking speed mask the relationship between lateral trunk motion and knee pain [24]. This masking effect means that without the presence of these variables the relationship between lateral trunk motion and knee pain would be weaker, absent or inverse. These variables were chosen to control for confounding the relationship between lateral trunk motion and knee pain. The selection of independent variables was based on the literature and clinical experience. The variables muscle strength, maximum walking speed, duration of complaints and BMI were selected based on the results of previous studies [1,2,4-6,8-10,20-22]. Age and stiffness were selected based on clinical experience. We repeated the analysis with the former mentioned variables at the patient level with the most severe K&L disease severity knee as index knee for lateral trunk motion. Meaning that trunk motion was defined as the movement of the trunk in the direction of the knee with the highest K&L disease severity. The analysis showed similar results (data not shown). Our results are not in agreement with the study of Tanaka et al. [10]. No significant differences were found between normal, unilateral and bilateral knees in trunk motion. The difference can be explained by the difference in analyses. In our study multivariable regression analyses was used and in the study of Tanaka et al. the Wilcoxon signed rank-test was conducted for differences between involved and non-involved limbs. The analysis was at the knee level, whereas in our study the analysis was at the patient level. In future studies the direction of trunk motion in relation to the most painful knee should be considered.

As age increases, it is known that joint range of motion (ROM) decreases. Joint ROM in relation to age is most evident in the spine, in particular lateral trunk flexion and axial rotation [25]. These authors also found a significant difference in lateral trunk flexion between men and women, with women exhibiting a higher mobility. Although the assessment of lateral trunk motion was performed differently, our findings are in agreement with the findings reported by these authors [25].

In our study, self-reported stiffness was measured by the WOMAC questionnaire, i.e. we evaluated the patient's perceived stiffness. Self-reported stiffness can be defined as the perception of decreased ease in moving the knee joint [3,26]. In the WOMAC questionnaire, self-reported stiffness is measured by two questions; (i) how severe was your stiffness after you first rose in the morning, and (ii) how severe has your stiffness been after sitting or lying down or while resting later in the day? Our results show that a high score of self-reported stiffness was related to more lateral trunk motion. It can be speculated that patients with self-reported knee stiffness adjust their walking pattern by reducing flexion at their knee and thereby change their lateral trunk motion. Future studies are necessary to determine the influence of self-reported and observed knee stiffness on lateral trunk motion in patients with knee OA.

Hunt et al. (2008) reported walking speed to be correlated to trunk motion (r = -0.24;p = 0.05), indicating that patients who walk slower show higher lateral trunk motion. Our results showed that patients with high speed showed high lateral trunk motion. These opposing findings may be attributed to how walking speed was assessed. Hunt et al. assessed walking speed by taking the average of multiple gait cycles while walking at a comfortable self-selected speed. In our study, patients were instructed to walk as fast as possible five times continuously up and down a level 20-meter corridor. This variable was chosen as an independent variable in analyses for the reason that maximum walking speed during the 100 m test and walking speed during gait analysis were highly correlated (r = 0.74; p = 0.000). Although the means (SD) in m/s of our study and of Hunt el al. (2008) were comparable (1.06 (0.16) and 1.14 (0.28), respectively), the different assessment methods may explain the opposing results. Future studies are necessary to determine the influence of different walking speeds on lateral trunk motion in patients with knee OA.

It should be noted that sagittal and transversal trunk motion would increase as lateral trunk motion increases. No studies have been found that assess the relationship between movements in all 3 dimensions in relation to knee pain. Pelvic rotation and hip joint motion influence lateral trunk motion in knee OA patients [5], therefore, these parameters should be considered in future studies. It is also suggested that the strength of the abductor muscles of the hip is of influence on trunk motion [7]. Hip muscle strength was not assessed in our study, but could have been of influence on trunk motion, as well as knee pain [27]. In future studies it is important to measure lateral trunk motion in relation to abductor muscle strength.

## Conclusions

Pain is associated with lateral trunk motion. This association is weak and is influenced by age, gender, self-reported stiffness and maximum walking speed.

## Competing interests

The authors declare that they have no competing interests.

## Authors' contributions

JD conceived the study and led the co-ordination of the study. MvdE, MS, JH, JvdN, and JD assisted with protocol design. MvdE, MS and JD wrote the manuscript. DK designed the statistical analysis. MvdE, MS, JvdN and JH designed the biomechanical measures. MvdE and MS designed the biomechanical and physical impairment measures. All authors participated in the trial design, provided feedback on drafts of this paper, read, and approved the final manuscript.

## Pre-publication history

The pre-publication history for this paper can be accessed here:

http://www.biomedcentral.com/1471-2474/12/141/prepub
